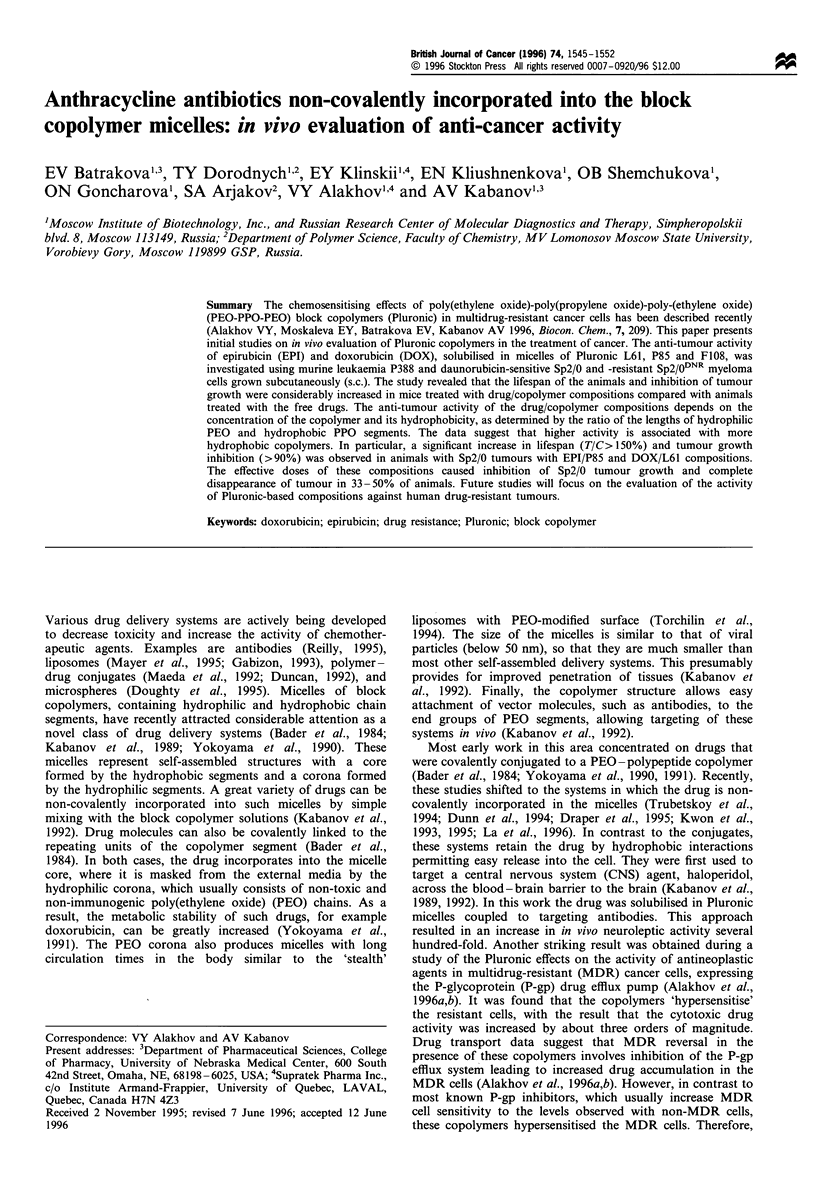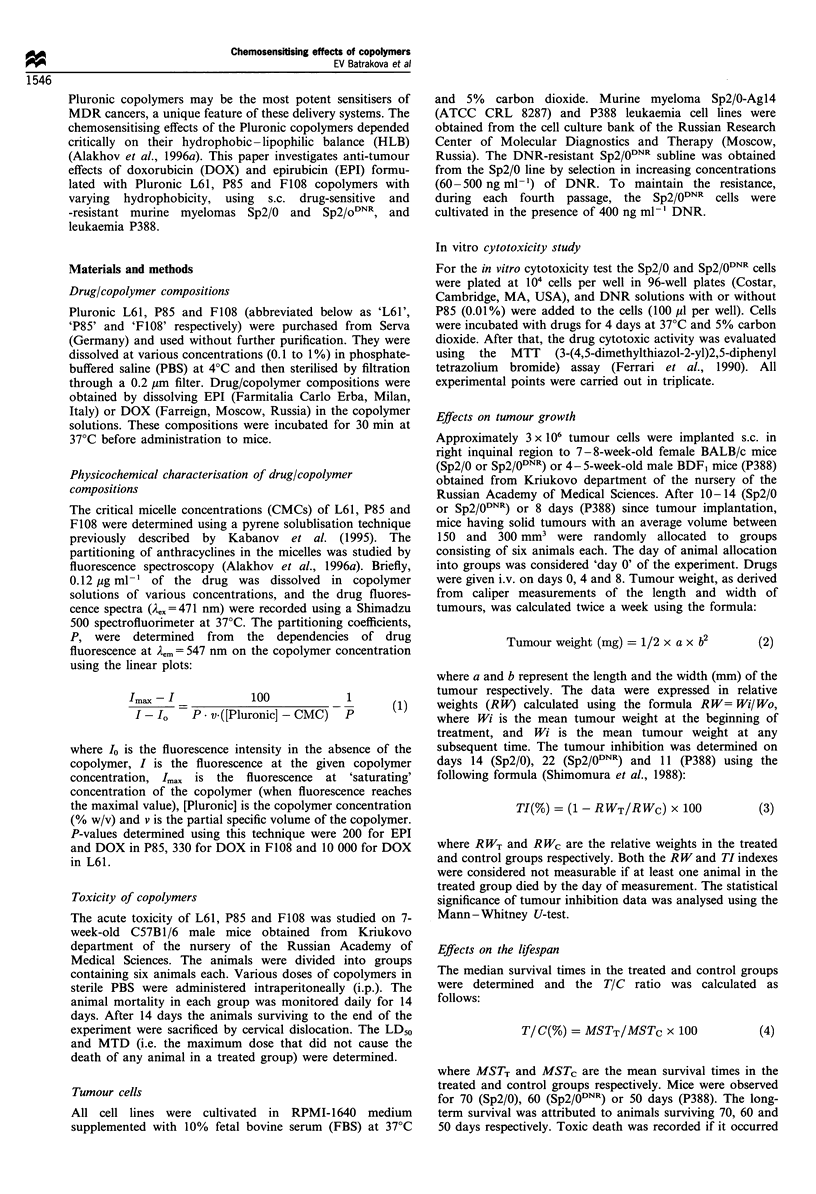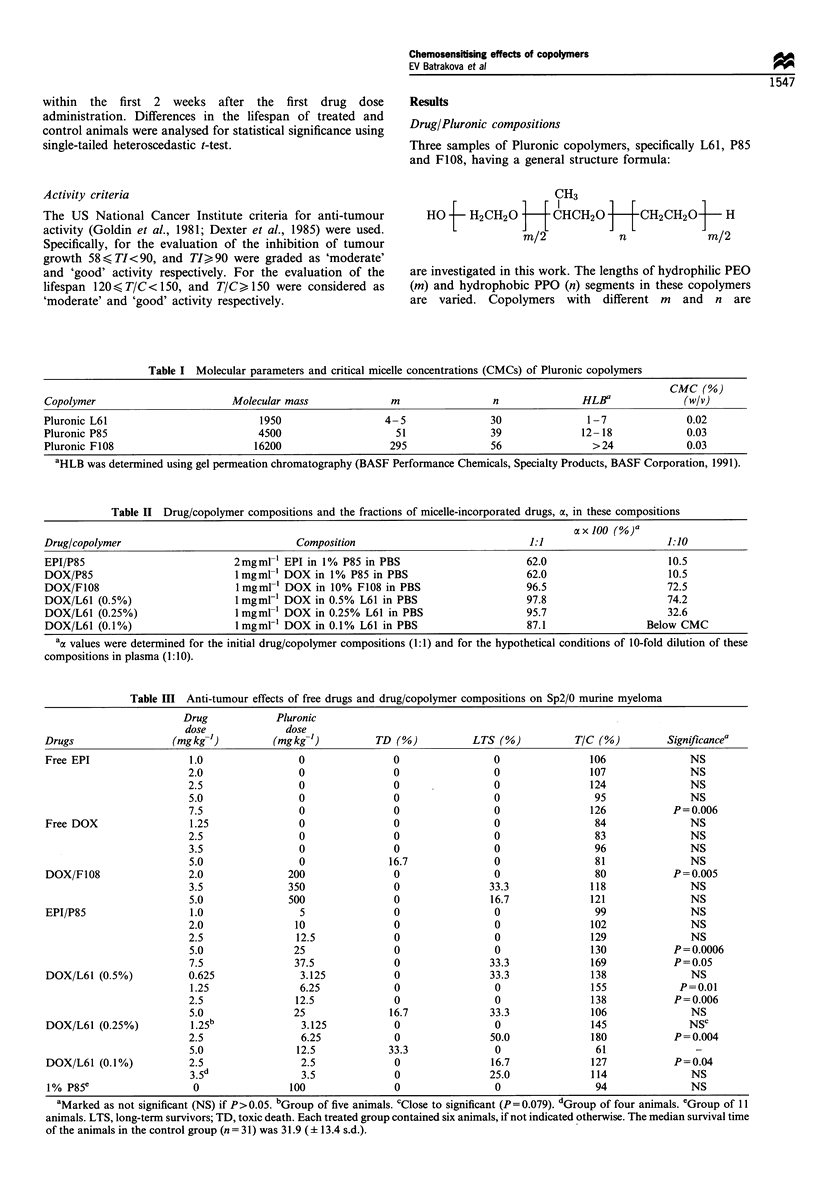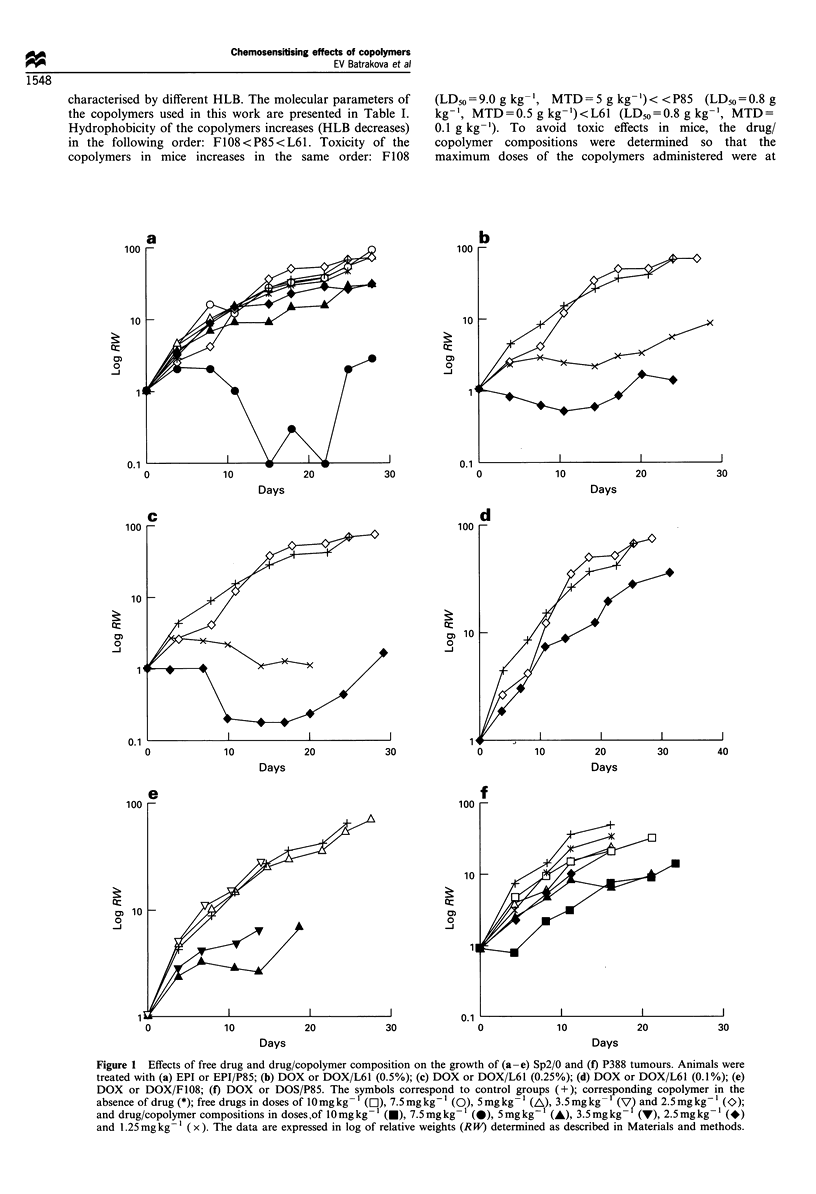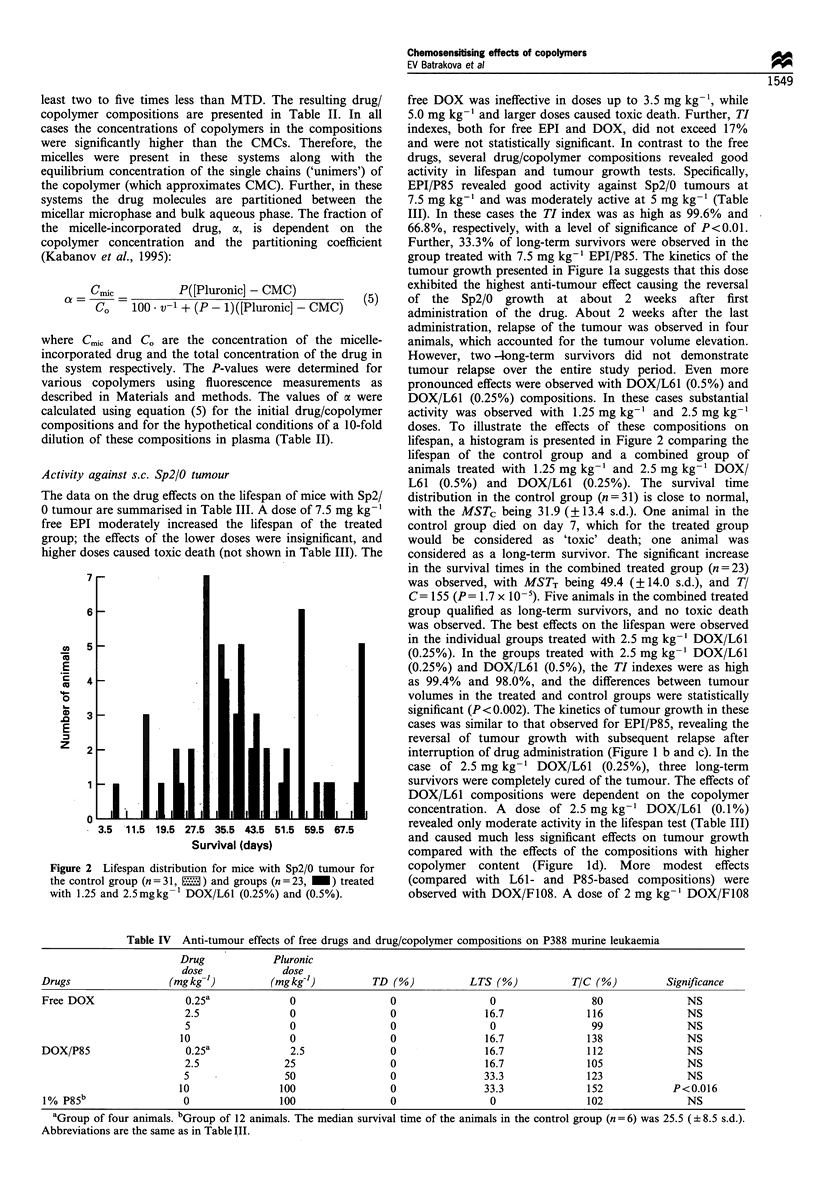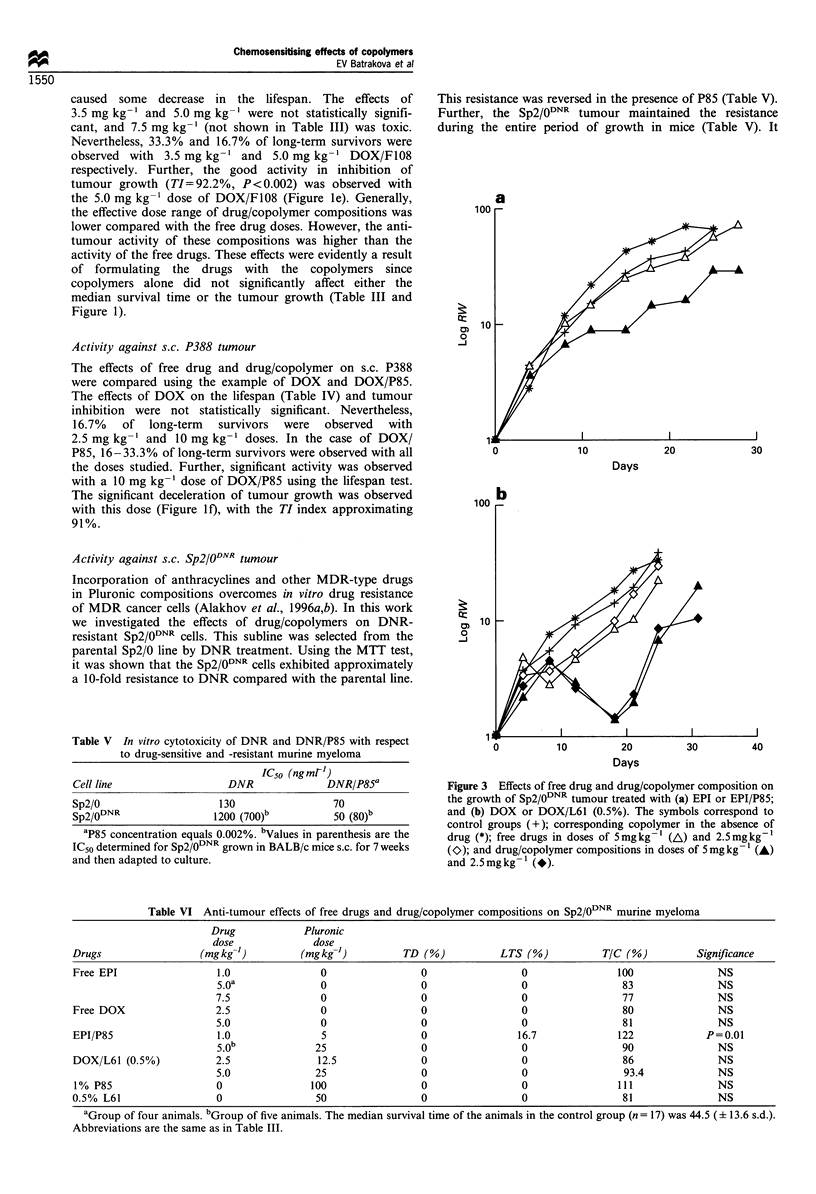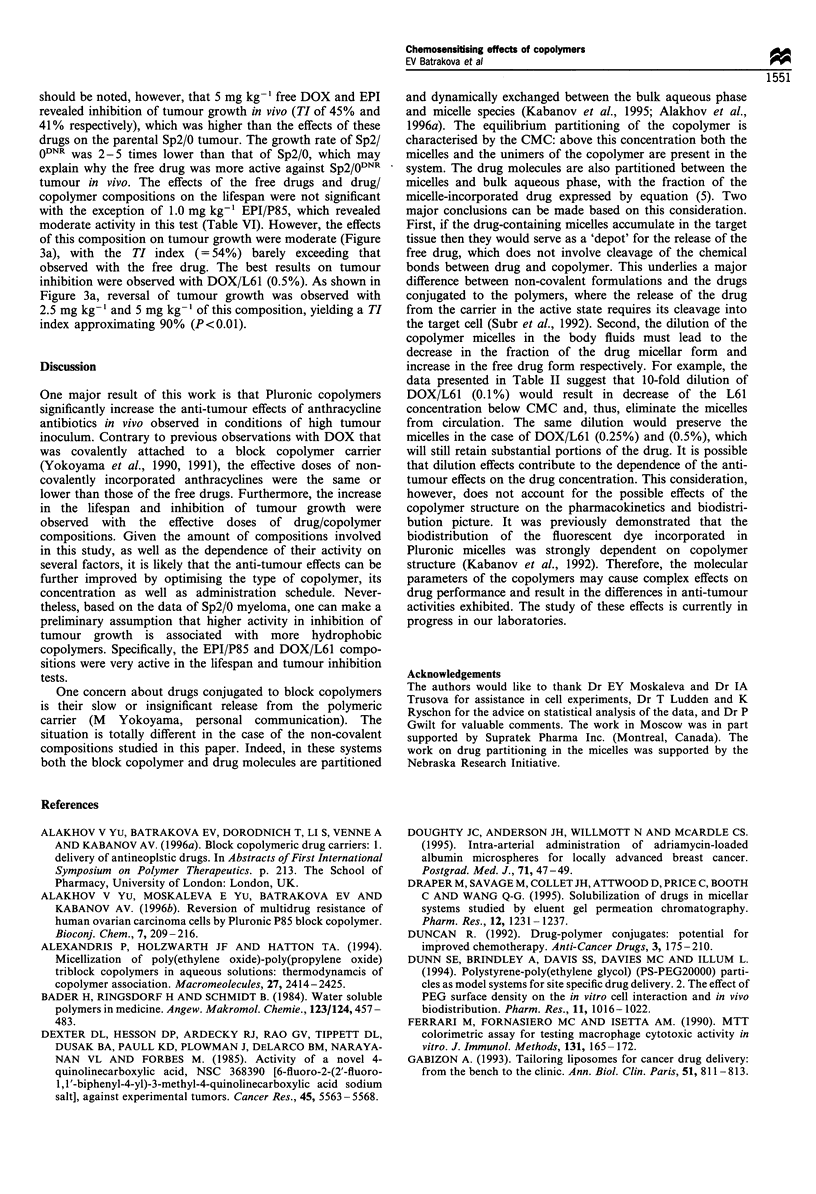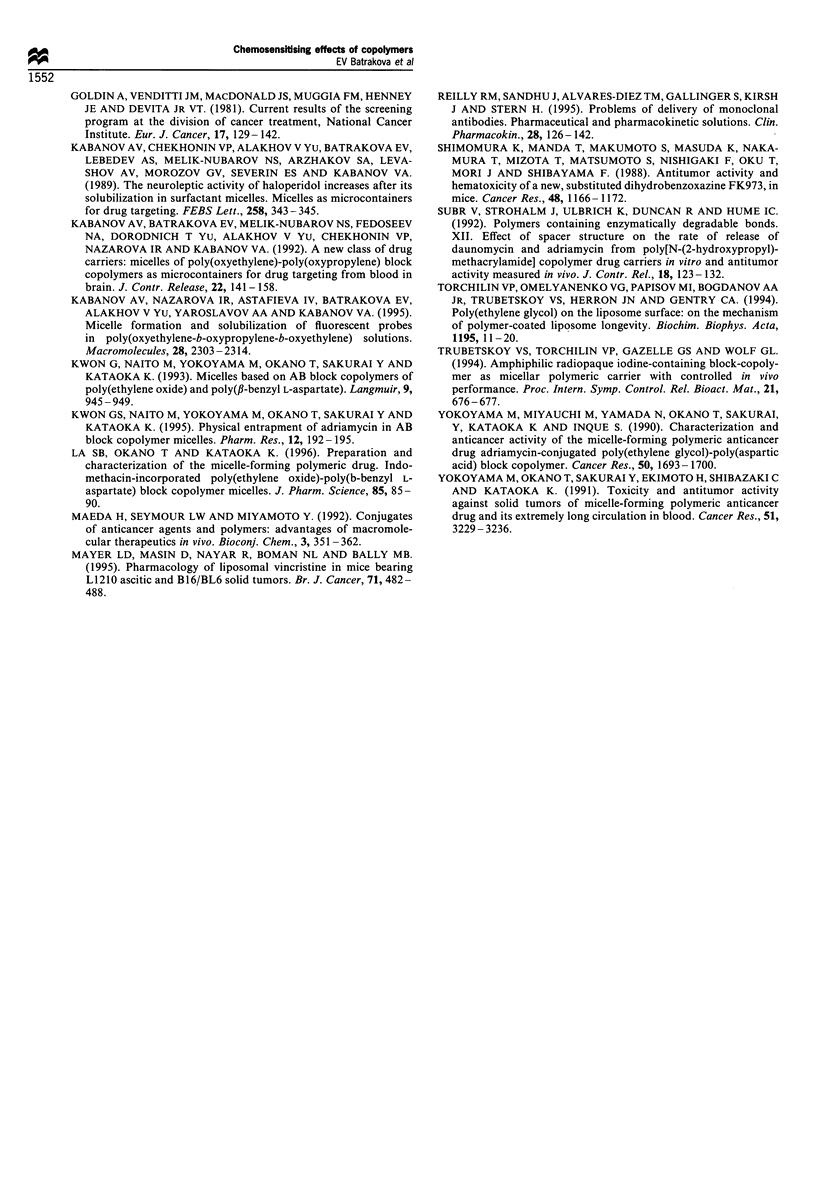# Anthracycline antibiotics non-covalently incorporated into the block copolymer micelles: in vivo evaluation of anti-cancer activity.

**DOI:** 10.1038/bjc.1996.587

**Published:** 1996-11

**Authors:** E. V. Batrakova, T. Y. Dorodnych, E. Y. Klinskii, E. N. Kliushnenkova, O. B. Shemchukova, O. N. Goncharova, S. A. Arjakov, V. Y. Alakhov, A. V. Kabanov

**Affiliations:** Moscow Institute of Biotechnology, Inc., and Russian Research Center of Molecular Diagnostics and Therapy.

## Abstract

The chemosensitising effects of poly(ethylene oxide)-poly(propylene oxide)-poly-(ethylene oxide) (PEO-PPO-PEO) block copolymers (Pluronic) in multidrug-resistant cancer cells has been described recently (Alakhov VY, Moskaleva EY, Batrakova EV, Kabanov AV 1996, Biocon. Chem., 7, 209). This paper presents initial studies on in vivo evaluation of Pluronic copolymers in the treatment of cancer. The anti-tumour activity of epirubicin (EPI) and doxorubicin (DOX), solubilised in micelles of Pluronic L61, P85 and F108, was investigated using murine leukaemia P388 and daunorubicin-sensitive Sp2/0 and -resistant Sp2/0(DNR) myeloma cells grown subcutaneously (s.c.). The study revealed that the lifespan of the animals and inhibition of tumour growth were considerably increased in mice treated with drug/copolymer compositions compared with animals treated with the free drugs. The anti-tumour activity of the drug/copolymer compositions depends on the concentration of the copolymer and its hydrophobicity, as determined by the ratio of the lengths of hydrophilic PEO and hydrophobic PPO segments. The data suggest that higher activity is associated with more hydrophobic copolymers. In particular, a significant increase in lifespan (T/C> 150%) and tumour growth inhibition (> 90%) was observed in animals with Sp2/0 tumours with EPI/P85 and DOX/L61 compositions. The effective doses of these compositions caused inhibition of Sp2/0 tumour growth and complete disappearance of tumour in 33-50% of animals. Future studies will focus on the evaluation of the activity of Pluronic-based compositions against human drug-resistant tumours.


British Journal of Cancer (1996) 74, 1545–1552
© 1996 Stockton Press All rights reserved 0007–0920/96 $12.00

### Anthracycline antibiotics non-covalently incorporated into the block copolymer micelles: *in vivo* evaluation of anti-cancer activity

EV Batrakova[1,3], TY Dorodnych[1,2], EY Klinskii[1,4], EN Kliushnenkova[1], OB Shemchukova[1], ON Goncharova[1], SA Arjakov[2], VY Alakhov[1,4] and AV Kabanov[1,3]

[1]*Moscow Institute of Biotechnology, Inc., and Russian Research Center of Molecular Diagnostics and Therapy, Simpheropolskii blvd. 8, Moscow 113149, Russia;* [2]*Department of Polymer Science, Faculty of Chemistry, MV Lomonosov Moscow State University, Vorobievy Gory, Moscow 119899 GSP, Russia.*

**Summary** The chemosensitising effects of poly(ethylene oxide)-poly(propylene oxide)-poly-(ethylene oxide) (PEO-PPO-PEO) block copolymers (Pluronic) in multidrug-resistant cancer cells has been described recently (Alakhov VY, Moskaleva EY, Batrakova EV, Kabanov AV 1996, *Biocon. Chem.*, **7**, 209). This paper presents initial studies on *in vivo* evaluation of Pluronic copolymers in the treatment of cancer. The anti-tumour activity of epirubicin (EPI) and doxorubicin (DOX), solubilised in micelles of Pluronic L61, P85 and F108, was investigated using murine leukaemia P388 and daunorubicin-sensitive Sp2/0 and -resistant Sp2/0$^{DNR}$ myeloma cells grown subcutaneously (s.c.). The study revealed that the lifespan of the animals and inhibition of tumour growth were considerably increased in mice treated with drug/copolymer compositions compared with animals treated with the free drugs. The anti-tumour activity of the drug/copolymer compositions depends on the concentration of the copolymer and its hydrophobicity, as determined by the ratio of the lengths of hydrophilic PEO and hydrophobic PPO segments. The data suggest that higher activity is associated with more hydrophobic copolymers. In particular, a significant increase in lifespan ($T/C>150\%$) and tumour growth inhibition (>90%) was observed in animals with Sp2/0 tumours with EPI/P85 and DOX/L61 compositions. The effective doses of these compositions caused inhibition of Sp2/0 tumour growth and complete disappearance of tumour in 33–50% of animals. Future studies will focus on the evaluation of the activity of Pluronic-based compositions against human drug-resistant tumours.

**Keywords:** doxorubicin; epirubicin; drug resistance; Pluronic; block copolymer

Various drug delivery systems are actively being developed to decrease toxicity and increase the activity of chemotherapeutic agents. Examples are antibodies (Reilly, 1995), liposomes (Mayer *et al.*, 1995; Gabizon, 1993), polymer–drug conjugates (Maeda *et al.*, 1992; Duncan, 1992), and microspheres (Doughty *et al.*, 1995). Micelles of block copolymers, containing hydrophilic and hydrophobic chain segments, have recently attracted considerable attention as a novel class of drug delivery systems (Bader *et al.*, 1984; Kabanov *et al.*, 1989; Yokoyama *et al.*, 1990). These micelles represent self-assembled structures with a core formed by the hydrophobic segments and a corona formed by the hydrophilic segments. A great variety of drugs can be non-covalently incorporated into such micelles by simple mixing with the block copolymer solutions (Kabanov *et al.*, 1992). Drug molecules can also be covalently linked to the repeating units of the copolymer segment (Bader *et al.*, 1984). In both cases, the drug incorporates into the micelle core, where it is masked from the external media by the hydrophilic corona, which usually consists of non-toxic and non-immunogenic poly(ethylene oxide) (PEO) chains. As a result, the metabolic stability of such drugs, for example doxorubicin, can be greatly increased (Yokoyama *et al.*, 1991). The PEO corona also produces micelles with long circulation times in the body similar to the 'stealth' liposomes with PEO-modified surface (Torchilin *et al.*, 1994). The size of the micelles is similar to that of viral particles (below 50 nm), so that they are much smaller than most other self-assembled delivery systems. This presumably provides for improved penetration of tissues (Kabanov *et al.*, 1992). Finally, the copolymer structure allows easy attachment of vector molecules, such as antibodies, to the end groups of PEO segments, allowing targeting of these systems *in vivo* (Kabanov *et al.*, 1992).

Most early work in this area concentrated on drugs that were covalently conjugated to a PEO–polypeptide copolymer (Bader *et al.*, 1984; Yokoyama *et al.*, 1990, 1991). Recently, these studies shifted to the systems in which the drug is non-covalently incorporated in the micelles (Trubetskoy *et al.*, 1994; Dunn *et al.*, 1994; Draper *et al.*, 1995; Kwon *et al.*, 1993, 1995; La *et al.*, 1996). In contrast to the conjugates, these systems retain the drug by hydrophobic interactions permitting easy release into the cell. They were first used to target a central nervous system (CNS) agent, haloperidol, across the blood–brain barrier to the brain (Kabanov *et al.*, 1989, 1992). In this work the drug was solubilised in Pluronic micelles coupled to targeting antibodies. This approach resulted in an increase in *in vivo* neuroleptic activity several hundred-fold. Another striking result was obtained during a study of the Pluronic effects on the activity of antineoplastic agents in multidrug-resistant (MDR) cancer cells, expressing the P-glycoprotein (P-gp) drug efflux pump (Alakhov *et al.*, 1996*a,b*). It was found that the copolymers 'hypersensitise' the resistant cells, with the result that the cytotoxic drug activity was increased by about three orders of magnitude. Drug transport data suggest that MDR reversal in the presence of these copolymers involves inhibition of the P-gp efflux system leading to increased drug accumulation in the MDR cells (Alakhov *et al.*, 1996*a,b*). However, in contrast to most known P-gp inhibitors, which usually increase MDR cell sensitivity to the levels observed with non-MDR cells, these copolymers hypersensitised the MDR cells. Therefore,

Correspondence: VY Alakhov and AV Kabanov
Present addresses: [3]Department of Pharmaceutical Sciences, College of Pharmacy, University of Nebraska Medical Center, 600 South 42nd Street, Omaha, NE, 68198–6025, USA; [4]Supratek Pharma Inc., c/o Institute Armand-Frappier, University of Quebec, LAVAL, Quebec, Canada H7N 4Z3
Received 2 November 1995; revised 7 June 1996; accepted 12 June 1996

Pluronic copolymers may be the most potent sensitisers of MDR cancers, a unique feature of these delivery systems. The chemosensitising effects of the Pluronic copolymers depended critically on their hydrophobic–lipophilic balance (HLB) (Alakhov *et al.*, 1996*a*). This paper investigates anti-tumour effects of doxorubicin (DOX) and epirubicin (EPI) formulated with Pluronic L61, P85 and F108 copolymers with varying hydrophobicity, using s.c. drug-sensitive and -resistant murine myelomas Sp2/0 and Sp2/o$^{DNR}$, and leukaemia P388.

### Materials and methods

### *Drug/copolymer compositions*

Pluronic L61, P85 and F108 (abbreviated below as 'L61', 'P85' and 'F108' respectively) were purchased from Serva (Germany) and used without further purification. They were dissolved at various concentrations (0.1 to 1%) in phosphate-buffered saline (PBS) at 4°C and then sterilised by filtration through a 0.2 $\mu$m filter. Drug/copolymer compositions were obtained by dissolving EPI (Farmitalia Carlo Erba, Milan, Italy) or DOX (Farreign, Moscow, Russia) in the copolymer solutions. These compositions were incubated for 30 min at 37°C before administration to mice.

### *Physicochemical characterisation of drug/copolymer compositions*

The critical micelle concentrations (CMCs) of L61, P85 and F108 were determined using a pyrene solubilisation technique previously described by Kabanov *et al.* (1995). The partitioning of anthracyclines in the micelles was studied by fluorescence spectroscopy (Alakhov *et al.*, 1996*a*). Briefly, 0.12 $\mu$g ml$^{-1}$ of the drug was dissolved in copolymer solutions of various concentrations, and the drug fluorescence spectra ($\lambda_{ex}=471$ nm) were recorded using a Shimadzu 500 spectrofluorimeter at 37°C. The partitioning coefficients, *P*, were determined from the dependencies of drug fluorescence at $\lambda_{em}=547$ nm on the copolymer concentration using the linear plots:

$$\frac{I_{\max}-I}{I-I_{o}}=\frac{100}{P\cdot v\cdot([\text{Pluronic}]-\text{CMC})}-\frac{1}{P} \qquad (1)$$

where $I_0$ is the fluorescence intensity in the absence of the copolymer, $I$ is the fluorescence at the given copolymer concentration, $I_{max}$ is the fluorescence at 'saturating' concentration of the copolymer (when fluorescence reaches the maximal value), [Pluronic] is the copolymer concentration (% w/v) and $v$ is the partial specific volume of the copolymer. *P*-values determined using this technique were 200 for EPI and DOX in P85, 330 for DOX in F108 and 10 000 for DOX in L61.

### *Toxicity of copolymers*

The acute toxicity of L61, P85 and F108 was studied on 7-week-old C57Bl/6 male mice obtained from Kriukovo department of the nursery of the Russian Academy of Medical Sciences. The animals were divided into groups containing six animals each. Various doses of copolymers in sterile PBS were administered intraperitoneally (i.p.). The animal mortality in each group was monitored daily for 14 days. After 14 days the animals surviving to the end of the experiment were sacrificed by cervical dislocation. The $LD_{50}$ and MTD (i.e. the maximum dose that did not cause the death of any animal in a treated group) were determined.

### *Tumour cells*

All cell lines were cultivated in RPMI-1640 medium supplemented with 10% fetal bovine serum (FBS) at 37°C and 5% carbon dioxide. Murine myeloma Sp2/0-Ag14 (ATCC CRL 8287) and P388 leukaemia cell lines were obtained from the cell culture bank of the Russian Research Center of Molecular Diagnostics and Therapy (Moscow, Russia). The DNR-resistant Sp2/0$^{DNR}$ subline was obtained from the Sp2/0 line by selection in increasing concentrations (60–500 ng ml$^{-1}$) of DNR. To maintain the resistance, during each fourth passage, the Sp2/0$^{DNR}$ cells were cultivated in the presence of 400 ng ml$^{-1}$ DNR.

### In vitro *cytotoxicity study*

For the *in vitro* cytotoxicity test the Sp2/0 and Sp2/0$^{DNR}$ cells were plated at $10^4$ cells per well in 96-well plates (Costar, Cambridge, MA, USA), and DNR solutions with or without P85 (0.01%) were added to the cells (100 $\mu$l per well). Cells were incubated with drugs for 4 days at 37°C and 5% carbon dioxide. After that, the drug cytotoxic activity was evaluated using the MTT (3-(4,5-dimethylthiazol-2-yl)2,5-diphenyl tetrazolium bromide) assay (Ferrari *et al.*, 1990). All experimental points were carried out in triplicate.

### *Effects on tumour growth*

Approximately $3\times10^6$ tumour cells were implanted s.c. in right inquinal region to 7–8-week-old female BALB/c mice (Sp2/0 or Sp2/0$^{DNR}$) or 4–5-week-old male $BDF_1$ mice (P388) obtained from Kriukovo department of the nursery of the Russian Academy of Medical Sciences. After 10–14 (Sp2/0 or Sp2/0$^{DNR}$) or 8 days (P388) since tumour implantation, mice having solid tumours with an average volume between 150 and 300 mm$^3$ were randomly allocated to groups consisting of six animals each. The day of animal allocation into groups was considered 'day 0' of the experiment. Drugs were given i.v. on days 0, 4 and 8. Tumour weight, as derived from caliper measurements of the length and width of tumours, was calculated twice a week using the formula:

$$\text{Tumour weight (mg)} = 1/2 \times a \times b^2 \qquad (2)$$

where $a$ and $b$ represent the length and the width (mm) of the tumour respectively. The data were expressed in relative weights (*RW*) calculated using the formula $RW=Wi/Wo$, where $Wi$ is the mean tumour weight at the beginning of treatment, and $Wi$ is the mean tumour weight at any subsequent time. The tumour inhibition was determined on days 14 (Sp2/0), 22 (Sp2/0$^{DNR}$) and 11 (P388) using the following formula (Shimomura *et al.*, 1988):

$$TI(\%) = (1 - RW_T/RW_C) \times 100 \qquad (3)$$

where $RW_T$ and $RW_C$ are the relative weights in the treated and control groups respectively. Both the *RW* and *TI* indexes were considered not measurable if at least one animal in the treated group died by the day of measurement. The statistical significance of tumour inhibition data was analysed using the Mann–Whitney *U*-test.

### *Effects on the lifespan*

The median survival times in the treated and control groups were determined and the *T/C* ratio was calculated as follows:

$$T/C(\%) = MST_T/MST_C \times 100 \qquad (4)$$

where $MST_T$ and $MST_C$ are the mean survival times in the treated and control groups respectively. Mice were observed for 70 (Sp2/0), 60 (Sp2/0$^{DNR}$) or 50 days (P388). The long-term survival was attributed to animals surviving 70, 60 and 50 days respectively. Toxic death was recorded if it occurred within the first 2 weeks after the first drug dose administration. Differences in the lifespan of treated and control animals were analysed for statistical significance using single-tailed heteroscedastic *t*-test.

*Activity criteria*

The US National Cancer Institute criteria for anti-tumour activity (Goldin *et al.*, 1981; Dexter *et al.*, 1985) were used. Specifically, for the evaluation of the inhibition of tumour growth $58 \leqslant TI < 90$, and $TI \geqslant 90$ were graded as 'moderate' and 'good' activity respectively. For the evaluation of the lifespan $120 \leqslant T/C < 150$, and $T/C \geqslant 150$ were considered as 'moderate' and 'good' activity respectively.

### Results

*Drug/Pluronic compositions*

Three samples of Pluronic copolymers, specifically L61, P85 and F108, having a general structure formula:

are investigated in this work. The lengths of hydrophilic PEO (*m*) and hydrophobic PPO (*n*) segments in these copolymers are varied. Copolymers with different *m* and *n* are

**Table I** Molecular parameters and critical micelle concentrations (CMCs) of Pluronic copolymers

| *Copolymer* | *Molecular mass* | *m* | *n* | *HLB*[a] | *CMC (%) (w/v)* |
|---|---|---|---|---|---|
| Pluronic L61 | 1950 | 4-5 | 30 | 1-7 | 0.02 |
| Pluronic P85 | 4500 | 51 | 39 | 12-18 | 0.03 |
| Pluronic F108 | 16200 | 295 | 56 | >24 | 0.03 |

[a]HLB was determined using gel permeation chromatography (BASF Performance Chemicals, Specialty Products, BASF Corporation, 1991).

**Table II** Drug/copolymer compositions and the fractions of micelle-incorporated drugs, α, in these compositions

| *Drug/copolymer* | *Composition* | *α × 100 (%)*[a] *1:1* | *1:10* |
|---|---|---|---|
| EPI/P85 | 2 mg ml$^{-1}$ EPI in 1% P85 in PBS | 62.0 | 10.5 |
| DOX/P85 | 1 mg ml$^{-1}$ DOX in 1% P85 in PBS | 62.0 | 10.5 |
| DOX/F108 | 1 mg ml$^{-1}$ DOX in 10% F108 in PBS | 96.5 | 72.5 |
| DOX/L61 (0.5%) | 1 mg ml$^{-1}$ DOX in 0.5% L61 in PBS | 97.8 | 74.2 |
| DOX/L61 (0.25%) | 1 mg ml$^{-1}$ DOX in 0.25% L61 in PBS | 95.7 | 32.6 |
| DOX/L61 (0.1%) | 1 mg ml$^{-1}$ DOX in 0.1% L61 in PBS | 87.1 | Below CMC |

[a]α values were determined for the initial drug/copolymer compositions (1:1) and for the hypothetical conditions of 10-fold dilution of these compositions in plasma (1:10).

**Table III** Anti-tumour effects of free drugs and drug/copolymer compositions on Sp2/0 murine myeloma

| *Drugs* | *Drug dose (mg kg$^{-1}$)* | *Pluronic dose (mg kg$^{-1}$)* | *TD (%)* | *LTS (%)* | *T/C (%)* | *Significance*[a] |
|---|---|---|---|---|---|---|
| Free EPI | 1.0 | 0 | 0 | 0 | 106 | NS |
| | 2.0 | 0 | 0 | 0 | 107 | NS |
| | 2.5 | 0 | 0 | 0 | 124 | NS |
| | 5.0 | 0 | 0 | 0 | 95 | NS |
| | 7.5 | 0 | 0 | 0 | 126 | $P=0.006$ |
| Free DOX | 1.25 | 0 | 0 | 0 | 84 | NS |
| | 2.5 | 0 | 0 | 0 | 83 | NS |
| | 3.5 | 0 | 0 | 0 | 96 | NS |
| | 5.0 | 0 | 16.7 | 0 | 81 | NS |
| DOX/F108 | 2.0 | 200 | 0 | 0 | 80 | $P=0.005$ |
| | 3.5 | 350 | 0 | 33.3 | 118 | NS |
| | 5.0 | 500 | 0 | 16.7 | 121 | NS |
| EPI/P85 | 1.0 | 5 | 0 | 0 | 99 | NS |
| | 2.0 | 10 | 0 | 0 | 102 | NS |
| | 2.5 | 12.5 | 0 | 0 | 129 | NS |
| | 5.0 | 25 | 0 | 0 | 130 | $P=0.0006$ |
| | 7.5 | 37.5 | 0 | 33.3 | 169 | $P=0.05$ |
| DOX/L61 (0.5%) | 0.625 | 3.125 | 0 | 33.3 | 138 | NS |
| | 1.25 | 6.25 | 0 | 0 | 155 | $P=0.01$ |
| | 2.5 | 12.5 | 0 | 0 | 138 | $P=0.006$ |
| | 5.0 | 25 | 16.7 | 33.3 | 106 | NS |
| DOX/L61 (0.25%) | 1.25[b] | 3.125 | 0 | 0 | 145 | NS[c] |
| | 2.5 | 6.25 | 0 | 50.0 | 180 | $P=0.004$ |
| | 5.0 | 12.5 | 33.3 | 0 | 61 | – |
| DOX/L61 (0.1%) | 2.5 | 2.5 | 0 | 16.7 | 127 | $P=0.04$ |
| | 3.5[d] | 3.5 | 0 | 25.0 | 114 | NS |
| 1% P85[e] | 0 | 100 | 0 | 0 | 94 | NS |

[a]Marked as not significant (NS) if $P>0.05$. [b]Group of five animals. [c]Close to significant ($P=0.079$). [d]Group of four animals. [e]Group of 11 animals. LTS, long-term survivors; TD, toxic death. Each treated group contained six animals, if not indicated otherwise. The median survival time of the animals in the control group ($n=31$) was 31.9 (± 13.4 s.d.).

characterised by different HLB. The molecular parameters of the copolymers used in this work are presented in Table I. Hydrophobicity of the copolymers increases (HLB decreases) in the following order: F108<P85<L61. Toxicity of the copolymers in mice increases in the same order: F108 ($LD_{50}$=9.0 g $kg^{-1}$, MTD=5 g $kg^{-1}$)<<P85 ($LD_{50}$=0.8 g $kg^{-1}$, MTD=0.5 g $kg^{-1}$)<L61 ($LD_{50}$=0.8 g $kg^{-1}$, MTD=0.1 g $kg^{-1}$). To avoid toxic effects in mice, the drug/copolymer compositions were determined so that the maximum doses of the copolymers administered were at

**Figure 1** Effects of free drug and drug/copolymer composition on the growth of (**a**–**e**) Sp2/0 and (**f**) P388 tumours. Animals were treated with (**a**) EPI or EPI/P85; (**b**) DOX or DOX/L61 (0.5%); (**c**) DOX or DOX/L61 (0.25%); (**d**) DOX or DOX/L61 (0.1%); (**e**) DOX or DOX/F108; (**f**) DOX or DOS/P85. The symbols correspond to control groups (+); corresponding copolymer in the absence of drug (*); free drugs in doses of 10 mg $kg^{-1}$ (□), 7.5 mg $kg^{-1}$ (○), 5 mg $kg^{-1}$ (△), 3.5 mg $kg^{-1}$ (▽) and 2.5 mg $kg^{-1}$ (◇); and drug/copolymer compositions in doses of 10 mg $kg^{-1}$ (■), 7.5 mg $kg^{-1}$ (●), 5 mg $kg^{-1}$ (▲), 3.5 mg $kg^{-1}$ (▼), 2.5 mg $kg^{-1}$ (◆) and 1.25 mg $kg^{-1}$ (×). The data are expressed in log of relative weights (*RW*) determined as described in Materials and methods.

least two to five times less than MTD. The resulting drug/copolymer compositions are presented in Table II. In all cases the concentrations of copolymers in the compositions were significantly higher than the CMCs. Therefore, the micelles were present in these systems along with the equilibrium concentration of the single chains ('unimers') of the copolymer (which approximates CMC). Further, in these systems the drug molecules are partitioned between the micellar microphase and bulk aqueous phase. The fraction of the micelle-incorporated drug, α, is dependent on the copolymer concentration and the partitioning coefficient (Kabanov *et al.*, 1995):

$$\alpha = \frac{C_{mic}}{C_o} = \frac{P([\text{Pluronic}] - \text{CMC})}{100 \cdot v^{-1} + (P-1)([\text{Pluronic}] - \text{CMC})} \quad (5)$$

where $C_{mic}$ and $C_o$ are the concentration of the micelle-incorporated drug and the total concentration of the drug in the system respectively. The *P*-values were determined for various copolymers using fluorescence measurements as described in Materials and methods. The values of α were calculated using equation (5) for the initial drug/copolymer compositions and for the hypothetical conditions of a 10-fold dilution of these compositions in plasma (Table II).

*Activity against s.c. Sp2/0 tumour*

The data on the drug effects on the lifespan of mice with Sp2/0 tumour are summarised in Table III. A dose of 7.5 mg kg$^{-1}$ free EPI moderately increased the lifespan of the treated group; the effects of the lower doses were insignificant, and higher doses caused toxic death (not shown in Table III). The

**Figure 2** Lifespan distribution for mice with Sp2/0 tumour for the control group ($n=31$, ▒) and groups ($n=23$, ■) treated with 1.25 and 2.5 mg kg$^{-1}$ DOX/L61 (0.25%) and (0.5%).

free DOX was ineffective in doses up to 3.5 mg kg$^{-1}$, while 5.0 mg kg$^{-1}$ and larger doses caused toxic death. Further, *TI* indexes, both for free EPI and DOX, did not exceed 17% and were not statistically significant. In contrast to the free drugs, several drug/copolymer compositions revealed good activity in lifespan and tumour growth tests. Specifically, EPI/P85 revealed good activity against Sp2/0 tumours at 7.5 mg kg$^{-1}$ and was moderately active at 5 mg kg$^{-1}$ (Table III). In these cases the *TI* index was as high as 99.6% and 66.8%, respectively, with a level of significance of $P<0.01$. Further, 33.3% of long-term survivors were observed in the group treated with 7.5 mg kg$^{-1}$ EPI/P85. The kinetics of the tumour growth presented in Figure 1a suggests that this dose exhibited the highest anti-tumour effect causing the reversal of the Sp2/0 growth at about 2 weeks after first administration of the drug. About 2 weeks after the last administration, relapse of the tumour was observed in four animals, which accounted for the tumour volume elevation. However, two long-term survivors did not demonstrate tumour relapse over the entire study period. Even more pronounced effects were observed with DOX/L61 (0.5%) and DOX/L61 (0.25%) compositions. In these cases substantial activity was observed with 1.25 mg kg$^{-1}$ and 2.5 mg kg$^{-1}$ doses. To illustrate the effects of these compositions on lifespan, a histogram is presented in Figure 2 comparing the lifespan of the control group and a combined group of animals treated with 1.25 mg kg$^{-1}$ and 2.5 mg kg$^{-1}$ DOX/L61 (0.5%) and DOX/L61 (0.25%). The survival time distribution in the control group ($n=31$) is close to normal, with the $MST_C$ being 31.9 (±13.4 s.d.). One animal in the control group died on day 7, which for the treated group would be considered as 'toxic' death; one animal was considered as a long-term survivor. The significant increase in the survival times in the combined treated group ($n=23$) was observed, with $MST_T$ being 49.4 (±14.0 s.d.), and $T/C=155$ ($P=1.7\times10^{-5}$). Five animals in the combined treated group qualified as long-term survivors, and no toxic death was observed. The best effects on the lifespan were observed in the individual groups treated with 2.5 mg kg$^{-1}$ DOX/L61 (0.25%). In the groups treated with 2.5 mg kg$^{-1}$ DOX/L61 (0.25%) and DOX/L61 (0.5%), the *TI* indexes were as high as 99.4% and 98.0%, and the differences between tumour volumes in the treated and control groups were statistically significant ($P<0.002$). The kinetics of tumour growth in these cases was similar to that observed for EPI/P85, revealing the reversal of tumour growth with subsequent relapse after interruption of drug administration (Figure 1 b and c). In the case of 2.5 mg kg$^{-1}$ DOX/L61 (0.25%), three long-term survivors were completely cured of the tumour. The effects of DOX/L61 compositions were dependent on the copolymer concentration. A dose of 2.5 mg kg$^{-1}$ DOX/L61 (0.1%) revealed only moderate activity in the lifespan test (Table III) and caused much less significant effects on tumour growth compared with the effects of the compositions with higher copolymer content (Figure 1d). More modest effects (compared with L61- and P85-based compositions) were observed with DOX/F108. A dose of 2 mg kg$^{-1}$ DOX/F108

**Table IV** Anti-tumour effects of free drugs and drug/copolymer compositions on P388 murine leukaemia

| *Drugs* | *Drug dose (mg kg$^{-1}$)* | *Pluronic dose (mg kg$^{-1}$)* | *TD (%)* | *LTS (%)* | *T/C (%)* | *Significance* |
|---|---|---|---|---|---|---|
| Free DOX | 0.25[a] | 0 | 0 | 0 | 80 | NS |
| | 2.5 | 0 | 0 | 16.7 | 116 | NS |
| | 5 | 0 | 0 | 0 | 99 | NS |
| | 10 | 0 | 0 | 16.7 | 138 | NS |
| DOX/P85 | 0.25[a] | 2.5 | 0 | 16.7 | 112 | NS |
| | 2.5 | 25 | 0 | 16.7 | 105 | NS |
| | 5 | 50 | 0 | 33.3 | 123 | NS |
| | 10 | 100 | 0 | 33.3 | 152 | $P<0.016$ |
| 1% P85[b] | 0 | 100 | 0 | 0 | 102 | NS |

[a]Group of four animals. [b]Group of 12 animals. The median survival time of the animals in the control group ($n=6$) was 25.5 (±8.5 s.d.). Abbreviations are the same as in Table III.

caused some decrease in the lifespan. The effects of 3.5 mg kg$^{-1}$ and 5.0 mg kg$^{-1}$ were not statistically significant, and 7.5 mg kg$^{-1}$ (not shown in Table III) was toxic. Nevertheless, 33.3% and 16.7% of long-term survivors were observed with 3.5 mg kg$^{-1}$ and 5.0 mg kg$^{-1}$ DOX/F108 respectively. Further, the good activity in inhibition of tumour growth ($TI$=92.2%, $P$<0.002) was observed with the 5.0 mg kg$^{-1}$ dose of DOX/F108 (Figure 1e). Generally, the effective dose range of drug/copolymer compositions was lower compared with the free drug doses. However, the antitumour activity of these compositions was higher than the activity of the free drugs. These effects were evidently a result of formulating the drugs with the copolymers since copolymers alone did not significantly affect either the median survival time or the tumour growth (Table III and Figure 1).

*Activity against s.c. P388 tumour*

The effects of free drug and drug/copolymer on s.c. P388 were compared using the example of DOX and DOX/P85. The effects of DOX on the lifespan (Table IV) and tumour inhibition were not statistically significant. Nevertheless, 16.7% of long-term survivors were observed with 2.5 mg kg$^{-1}$ and 10 mg kg$^{-1}$ doses. In the case of DOX/P85, 16–33.3% of long-term survivors were observed with all the doses studied. Further, significant activity was observed with a 10 mg kg$^{-1}$ dose of DOX/P85 using the lifespan test. The significant deceleration of tumour growth was observed with this dose (Figure 1f), with the $TI$ index approximating 91%.

*Activity against s.c. Sp2/0$^{DNR}$ tumour*

Incorporation of anthracyclines and other MDR-type drugs in Pluronic compositions overcomes *in vitro* drug resistance of MDR cancer cells (Alakhov *et al.*, 1996*a*,*b*). In this work we investigated the effects of drug/copolymers on DNR-resistant Sp2/0$^{DNR}$ cells. This subline was selected from the parental Sp2/0 line by DNR treatment. Using the MTT test, it was shown that the Sp2/0$^{DNR}$ cells exhibited approximately a 10-fold resistance to DNR compared with the parental line. This resistance was reversed in the presence of P85 (Table V). Further, the Sp2/0$^{DNR}$ tumour maintained the resistance during the entire period of growth in mice (Table V). It

**Table V** *In vitro* cytotoxicity of DNR and DNR/P85 with respect to drug-sensitive and -resistant murine myeloma

| | $IC_{50}$ (ng ml$^{-1}$) | |
|---|---|---|
| *Cell line* | *DNR* | *DNR/P85*[a] |
| Sp2/0 | 130 | 70 |
| Sp2/0$^{DNR}$ | 1200 (700)[b] | 50 (80)[b] |

[a]P85 concentration equals 0.002%. [b]Values in parenthesis are the $IC_{50}$ determined for Sp2/0$^{DNR}$ grown in BALB/c mice s.c. for 7 weeks and then adapted to culture.

**Figure 3** Effects of free drug and drug/copolymer composition on the growth of Sp2/0$^{DNR}$ tumour treated with (**a**) EPI or EPI/P85; and (**b**) DOX or DOX/L61 (0.5%). The symbols correspond to control groups (+); corresponding copolymer in the absence of drug (*); free drugs in doses of 5 mg kg$^{-1}$ (△) and 2.5 mg kg$^{-1}$ (◇); and drug/copolymer compositions in doses of 5 mg kg$^{-1}$ (▲) and 2.5 mg kg$^{-1}$ (◆).

**Table VI** Anti-tumour effects of free drugs and drug/copolymer compositions on Sp2/0$^{DNR}$ murine myeloma

| *Drugs* | *Drug dose (mg kg$^{-1}$)* | *Pluronic dose (mg kg$^{-1}$)* | *TD (%)* | *LTS (%)* | *T/C (%)* | *Significance* |
|---|---|---|---|---|---|---|
| Free EPI | 1.0 | 0 | 0 | 0 | 100 | NS |
| | 5.0[a] | 0 | 0 | 0 | 83 | NS |
| | 7.5 | 0 | 0 | 0 | 77 | NS |
| Free DOX | 2.5 | 0 | 0 | 0 | 80 | NS |
| | 5.0 | 0 | 0 | 0 | 81 | NS |
| EPI/P85 | 1.0 | 5 | 0 | 16.7 | 122 | $P$=0.01 |
| | 5.0[b] | 25 | 0 | 0 | 90 | NS |
| DOX/L61 (0.5%) | 2.5 | 12.5 | 0 | 0 | 86 | NS |
| | 5.0 | 25 | 0 | 0 | 93.4 | NS |
| 1% P85 | 0 | 100 | 0 | 0 | 111 | NS |
| 0.5% L61 | 0 | 50 | 0 | 0 | 81 | NS |

[a]Group of four animals. [b]Group of five animals. The median survival time of the animals in the control group ($n$=17) was 44.5 (±13.6 s.d.). Abbreviations are the same as in Table III.

should be noted, however, that 5 mg $kg^{-1}$ free DOX and EPI revealed inhibition of tumour growth *in vivo* (*TI* of 45% and 41% respectively), which was higher than the effects of these drugs on the parental Sp2/0 tumour. The growth rate of Sp2/$0^{DNR}$ was 2–5 times lower than that of Sp2/0, which may explain why the free drug was more active against Sp2/$0^{DNR}$ tumour *in vivo*. The effects of the free drugs and drug/copolymer compositions on the lifespan were not significant with the exception of 1.0 mg $kg^{-1}$ EPI/P85, which revealed moderate activity in this test (Table VI). However, the effects of this composition on tumour growth were moderate (Figure 3a), with the *TI* index (=54%) barely exceeding that observed with the free drug. The best results on tumour inhibition were observed with DOX/L61 (0.5%). As shown in Figure 3a, reversal of tumour growth was observed with 2.5 mg $kg^{-1}$ and 5 mg $kg^{-1}$ of this composition, yielding a *TI* index approximating 90% ($P<0.01$).

### Discussion

One major result of this work is that Pluronic copolymers significantly increase the anti-tumour effects of anthracycline antibiotics *in vivo* observed in conditions of high tumour inoculum. Contrary to previous observations with DOX that was covalently attached to a block copolymer carrier (Yokoyama *et al.*, 1990, 1991), the effective doses of non-covalently incorporated anthracyclines were the same or lower than those of the free drugs. Furthermore, the increase in the lifespan and inhibition of tumour growth were observed with the effective doses of drug/copolymer compositions. Given the amount of compositions involved in this study, as well as the dependence of their activity on several factors, it is likely that the anti-tumour effects can be further improved by optimising the type of copolymer, its concentration as well as administration schedule. Nevertheless, based on the data of Sp2/0 myeloma, one can make a preliminary assumption that higher activity in inhibition of tumour growth is associated with more hydrophobic copolymers. Specifically, the EPI/P85 and DOX/L61 compositions were very active in the lifespan and tumour inhibition tests.

One concern about drugs conjugated to block copolymers is their slow or insignificant release from the polymeric carrier (M Yokoyama, personal communication). The situation is totally different in the case of the non-covalent compositions studied in this paper. Indeed, in these systems both the block copolymer and drug molecules are partitioned and dynamically exchanged between the bulk aqueous phase and micelle species (Kabanov *et al.*, 1995; Alakhov *et al.*, 1996*a*). The equilibrium partitioning of the copolymer is characterised by the CMC: above this concentration both the micelles and the unimers of the copolymer are present in the system. The drug molecules are also partitioned between the micelles and bulk aqueous phase, with the fraction of the micelle-incorporated drug expressed by equation (5). Two major conclusions can be made based on this consideration. First, if the drug-containing micelles accumulate in the target tissue then they would serve as a 'depot' for the release of the free drug, which does not involve cleavage of the chemical bonds between drug and copolymer. This underlies a major difference between non-covalent formulations and the drugs conjugated to the polymers, where the release of the drug from the carrier in the active state requires its cleavage into the target cell (Subr *et al.*, 1992). Second, the dilution of the copolymer micelles in the body fluids must lead to the decrease in the fraction of the drug micellar form and increase in the free drug form respectively. For example, the data presented in Table II suggest that 10-fold dilution of DOX/L61 (0.1%) would result in decrease of the L61 concentration below CMC and, thus, eliminate the micelles from circulation. The same dilution would preserve the micelles in the case of DOX/L61 (0.25%) and (0.5%), which will still retain substantial portions of the drug. It is possible that dilution effects contribute to the dependence of the anti-tumour effects on the drug concentration. This consideration, however, does not account for the possible effects of the copolymer structure on the pharmacokinetics and biodistribution picture. It was previously demonstrated that the biodistribution of the fluorescent dye incorporated in Pluronic micelles was strongly dependent on copolymer structure (Kabanov *et al.*, 1992). Therefore, the molecular parameters of the copolymers may cause complex effects on drug performance and result in the differences in anti-tumour activities exhibited. The study of these effects is currently in progress in our laboratories.

**Acknowledgements**
The authors would like to thank Dr EY Moskaleva and Dr IA Trusova for assistance in cell experiments, Dr T Ludden and K Ryschon for the advice on statistical analysis of the data, and Dr P Gwilt for valuable comments. The work in Moscow was in part supported by Supratek Pharma Inc. (Montreal, Canada). The work on drug partitioning in the micelles was supported by the Nebraska Research Initiative.

### References

ALAKHOV V YU, BATRAKOVA EV, DORODNICH T, LI S, VENNE A AND KABANOV AV. (1996*a*). Block copolymeric drug carriers: 1. delivery of antineoplstic drugs. In *Abstracts of First International Symposium on Polymer Therapeutics*. p. 213. The School of Pharmacy, University of London: London, UK.

ALAKHOV V YU, MOSKALEVA E YU, BATRAKOVA EV AND KABANOV AV. (1996*b*). Reversion of multidrug resistance of human ovarian carcinoma cells by Pluronic P85 block copolymer. *Bioconj. Chem.*, **7**, 209–216.

ALEXANDRIS P, HOLZWARTH JF AND HATTON TA. (1994). Micellization of poly(ethylene oxide)-poly(propylene oxide) triblock copolymers in aqueous solutions: thermodynamcis of copolymer association. *Macromeolecules*, **27**, 2414–2425.

BADER H, RINGSDORF H AND SCHMIDT B. (1984). Water soluble polymers in medicine. *Angew. Makromol. Chemie.*, **123/124**, 457–483.

DEXTER DL, HESSON DP, ARDECKY RJ, RAO GV, TIPPETT DL, DUSAK BA, PAULL KD, PLOWMAN J, DELARCO BM, NARAYANAN VL AND FORBES M. (1985). Activity of a novel 4-quinolinecarboxylic acid, NSC 368390 [6-fluoro-2-(2′-fluoro-1,1′-biphenyl-4-yl)-3-methyl-4-quinolinecarboxylic acid sodium salt], against experimental tumors. *Cancer Res.*, **45**, 5563–5568.

DOUGHTY JC, ANDERSON JH, WILLMOTT N AND MCARDLE CS. (1995). Intra-arterial administration of adriamycin-loaded albumin microspheres for locally advanced breast cancer. *Postgrad. Med. J.*, **71**, 47–49.

DRAPER M, SAVAGE M, COLLET JH, ATTWOOD D, PRICE C, BOOTH C AND WANG Q-G. (1995). Solubilization of drugs in micellar systems studied by eluent gel permeation chromatography. *Pharm. Res.*, **12**, 1231–1237.

DUNCAN R. (1992). Drug-polymer conjugates: potential for improved chemotherapy. *Anti-Cancer Drugs*, **3**, 175–210.

DUNN SE, BRINDLEY A, DAVIS SS, DAVIES MC AND ILLUM L. (1994). Polystyrene-poly(ethylene glycol) (PS-PEG20000) particles as model systems for site specific drug delivery. 2. The effect of PEG surface density on the *in vitro* cell interaction and *in vivo* biodistribution. *Pharm. Res.*, **11**, 1016–1022.

FERRARI M, FORNASIERO MC AND ISETTA AM. (1990). MTT colorimetric assay for testing macrophage cytotoxic activity *in vitro*. *J. Immunol. Methods*, **131**, 165–172.

GABIZON A. (1993). Tailoring liposomes for cancer drug delivery: from the bench to the clinic. *Ann. Biol. Clin. Paris*, **51**, 811–813.

Goldin A, Venditti JM, MacDonald JS, Muggia FM, Henney JE and DeVita Jr VT. (1981). Current results of the screening program at the division of cancer treatment, National Cancer Institute. *Eur. J. Cancer*, **17**, 129–142.

Kabanov AV, Chekhonin VP, Alakhov V Yu, Batrakova EV, Lebedev AS, Melik-Nubarov NS, Arzhakov SA, Levashov AV, Morozov GV, Severin ES and Kabanov VA. (1989). The neuroleptic activity of haloperidol increases after its solubilization in surfactant micelles. Micelles as microcontainers for drug targeting. *FEBS Lett.*, **258**, 343–345.

Kabanov AV, Batrakova EV, Melik-Nubarov NS, Fedoseev NA, Dorodnich T Yu, Alakhov V Yu, Chekhonin VP, Nazarova IR and Kabanov VA. (1992). A new class of drug carriers: micelles of poly(oxyethylene)-poly(oxypropylene) block copolymers as microcontainers for drug targeting from blood in brain. *J. Contr. Release*, **22**, 141–158.

Kabanov AV, Nazarova IR, Astafieva IV, Batrakova EV, Alakhov V Yu, Yaroslavov AA and Kabanov VA. (1995). Micelle formation and solubilization of fluorescent probes in poly(oxyethylene-*b*-oxypropylene-*b*-oxyethylene) solutions. *Macromolecules*, **28**, 2303–2314.

Kwon G, Naito M, Yokoyama M, Okano T, Sakurai Y and Kataoka K. (1993). Micelles based on AB block copolymers of poly(ethylene oxide) and poly($\beta$-benzyl L-aspartate). *Langmuir*, **9**, 945–949.

Kwon GS, Naito M, Yokoyama M, Okano T, Sakurai Y and Kataoka K. (1995). Physical entrapment of adriamycin in AB block copolymer micelles. *Pharm. Res.*, **12**, 192–195.

La SB, Okano T and Kataoka K. (1996). Preparation and characterization of the micelle-forming polymeric drug. Indomethacin-incorporated poly(ethylene oxide)-poly(b-benzyl L-aspartate) block copolymer micelles. *J. Pharm. Science*, **85**, 85–90.

Maeda H, Seymour LW and Miyamoto Y. (1992). Conjugates of anticancer agents and polymers: advantages of macromolecular therapeutics *in vivo*. *Bioconj. Chem.*, **3**, 351–362.

Mayer LD, Masin D, Nayar R, Boman NL and Bally MB. (1995). Pharmacology of liposomal vincristine in mice bearing L1210 ascitic and B16/BL6 solid tumors. *Br. J. Cancer*, **71**, 482–488.

Reilly RM, Sandhu J, Alvares-Diez TM, Gallinger S, Kirsh J and Stern H. (1995). Problems of delivery of monoclonal antibodies. Pharmaceutical and pharmacokinetic solutions. *Clin. Pharmacokin.*, **28**, 126–142.

Shimomura K, Manda T, Makumoto S, Masuda K, Nakamura T, Mizota T, Matsumoto S, Nishigaki F, Oku T, Mori J and Shibayama F. (1988). Antitumor activity and hematoxicity of a new, substituted dihydrobenzoxazine FK973, in mice. *Cancer Res.*, **48**, 1166–1172.

Subr V, Strohalm J, Ulbrich K, Duncan R and Hume IC. (1992). Polymers containing enzymatically degradable bonds. XII. Effect of spacer structure on the rate of release of daunomycin and adriamycin from poly[N-(2-hydroxypropyl)-methacrylamide] copolymer drug carriers *in vitro* and antitumor activity measured *in vivo*. *J. Contr. Rel.*, **18**, 123–132.

Torchilin VP, Omelyanenko VG, Papisov MI, Bogdanov AA Jr, Trubetskoy VS, Herron JN and Gentry CA. (1994). Poly(ethylene glycol) on the liposome surface: on the mechanism of polymer-coated liposome longevity. *Biochim. Biophys. Acta*, **1195**, 11–20.

Trubetskoy VS, Torchilin VP, Gazelle GS and Wolf GL. (1994). Amphiphilic radiopaque iodine-containing block-copolymer as micellar polymeric carrier with controlled *in vivo* performance. *Proc. Intern. Symp. Control. Rel. Bioact. Mat.*, **21**, 676–677.

Yokoyama M, Miyauchi M, Yamada N, Okano T, Sakurai, Y, Kataoka K and Inque S. (1990). Characterization and anticancer activity of the micelle-forming polymeric anticancer drug adriamycin-conjugated poly(ethylene glycol)-poly(aspartic acid) block copolymer. *Cancer Res.*, **50**, 1693–1700.

Yokoyama M, Okano T, Sakurai Y, Ekimoto H, Shibazaki C and Kataoka K. (1991). Toxicity and antitumor activity against solid tumors of micelle-forming polymeric anticancer drug and its extremely long circulation in blood. *Cancer Res.*, **51**, 3229–3236.